# Supplementary material for: Identifying Risk Factors for Lower Reproductive Tract Infections among Women Using Reusable Absorbents in Odisha, India
Source: Int J Environ Res Public Health. 2021 Apr 29;18(9):4778. doi: 10.3390/ijerph18094778 (PMC8124764; doi:10.3390/ijerph18094778)
Supplement: Supplementary file 1 [file ijerph-18-04778-s001.zip › ijerph-1127873-supplementary.pdf]

Supplementary File 1

Study One Questionnaire

	Prompt	*Values*
ID	Participant ID	Unique integer
Location	Place where the sample was collected	1=Bhubaneswar<br>2=Rourkela
Date	Date of the interview	
1	How old are you?	
2	What is your marital status (Select one)	1= Single, never married<br>2 = Married<br>3 =Widowed<br>4 = Divorced<br>5 = Separated<br>6= Other
2bi.	How many years have you been married?	
3	Have you given birth within the last three months?	1=yes 0=no
4	How old were you when you had your first period?	
5	What is your religion?	1= Hindu<br>2= Muslim<br>3=Christian<br>4= Other
6	What is your caste or tribe?	1= Scheduled caste (SC)<br>2= Scheduled tribe (ST)<br>3 = Other backward caste (OBC)<br>4= Other caste
7	What is the highest standard year of education that you have completed?	1= No formal education<br>2= Some primary (1-4th year)<br>3 = Completed primary (5th year)<br>4= Some secondary (6-10th year)<br>5= Completed +2 year (12th year)<br>6 = Completed +3 year (university, etc.)
8	What is your occupation?	1= Employed or self-employed<br>2= Housewife<br>3= Student<br>4= Other
9	How many people live in your household?	
10	Which is the monthly household income (in rupees of all family members)?	1= Bellow 5,000<br>2= 5,000-10,000<br>3= 10,000-20,000<br>4= 20,000-30,000<br>5= 30,000 and above<br>6= Don't Know
11	Does your household own a BPL card? (please verify)	1=Yes Verified<br>2= Yes not Verified<br>0=No
12	Do you have abnormal vaginal discharge? (more abundant than normally)	1=yes 0=no
13	Do you have a feeling of burning or itching in the genitalia (vulvovaginal area)?	1=yes 0=no
14	Do you have genital sores?	1=yes 0=no
15	Do you feel the need to urinate more frequently	1=yes 0=no
16	Do you have a feeling of burning or itching when urinating?	1=yes 0=no
17	Do you have cloudy urine or blood in your urine	1=yes 0=no

18	Do you have pain in your lower back?	1=yes 0=no
19	Do you have pain in your belly or stomach?	1=yes 0=no
20	Do you have pain in your breast?	1=yes 0=no
21	Have you had hysterectomy before?	1=yes 0=no
22	Do you present abnormal vaginal bleeding out of the menstruation days?	1=yes 0=no
23	When was the last time that you had your period? Specify date :	
24	Do you use any type of Contraceptive method at the moment?	0=No<br>1=Yes<br>2=I Don't know
25	If you use a contraceptive method, choose which one?	1= Condom use<br>2= Injections or birth control pills<br>3= Intrauterine device<br>4= Tubal ligation<br>5= Other
26	Have you taken any antibiotic treatment during the last 2 weeks?	0=No<br>1=Yes<br>2=I Don't know
27	Is the problem why you are coming to the clinic a recurrent one (it is happening more than once)?	1=yes 0=no
28	How often these problems appeared in the last year approximately?	1= Every Months<br>2= 6 times in a year<br>3= 4 times in a year<br>4= 3 times in a year<br>5= Less than 3 times<br>6=I don't remember
29	Did you change your menstrual hygienic habits after having this recurrent symptom?	1=yes 0=no
30a.	If yes, did you change Menstrual absorbent type?	1=yes 0=no
30b.	If yes, did you change vaginal washing practices?	1=yes 0=no
30c.	If yes, did you change body washing practices?	1=yes 0=no
30d.	If yes, did you change absorbent washing practices (If applied)?	1=yes 0=no
30e.	If yes, did you change place for changing menstrual absorbent?	1=yes 0=no
30f.	If yes, did you change place to defecate or urinate every day?	1=yes 0=no
30g.	If yes, did you change place to defecate or urinate during menstruation?	1=yes 0=no
31	What absorbent material did you use most often during the last 6 cycles?	1= Disposable sanitary pads<br>2=Reusable cloths/towel<br>3= tampon<br>4= nothing<br>5= other
32	What type of reusable material do you use?	1=Old cotton fabric (sari or other)<br>2=Old silk/nylon fabric (sari or other)<br>3=Towel<br>4=Others
33	Why do you use this type of absorbent?	1=Because we have always used it at home<br>2=Because I prefer to use it<br>3=Because this is what I can afford<br>4=Because I cannot find another type of absorbent in any shop/place close to my house<br>5=Other
34	How often did you change the absorbent material in your heavier days?	1=Once a Day<br>2=Twice a Day<br>3=Three times a day<br>4= Other
35	Do you normally stay at home when menstruating?	1=yes 0=no

36	Where did you change your absorbent material?	1= In a household toilet<br>2= In a private room in the house<br>3= In a neighbors/relatives/public facility outside the house or yard<br>4= In the bush/field/ground/river/stream site<br>6 =Other
37	What to did you do with the absorbent material?	1= Dispose it<br>2 = Reuse it
38	If you reuse it, where did you wash it?	1= Inside the toilet stall.<br>2= In the public pond or river<br>3= At the tube well<br>4 = I don't wash it<br>5= Other……………..
39	How did you wash your sanitary cloths?	1= With water<br>2= With water and soap or detergent<br>3= With water and mud/ash<br>4= Other……………
40	After washing it, how did you dry the cloth?	1= Dry it in the sun or open space<br>2= Dry it inside the house.<br>3= I don't dry it.<br>4=Other
41	How did you store the cloth for use next time?	1= Wrapped in polythene<br>2= Wrapped in another material -<br>3= Wrapped in nothing<br>4=Other……….
42	Where do you store the cloth for use next time?	1= Within my clothes<br>2= In the toilet<br>3= In some place of the changing room.<br>4= Other…………
43	If you was not reuse it, where did you place it?	1= Inside latrine<br>2= In a rubbish bin inside or close to the latrine<br>3= In the household rubbish bin<br>4= Put it in the pond<br>5= I burn it.<br>6= Discard in any other open space.<br>7= Other……….
44	What type of washing (bath or vaginal wash) did you practice during Menstruation	1= Only vaginal wash.<br>2= Bath of full body.<br>3= I don't wash myself
45	How often did you wash yourself (bath or vaginal wash) during Menstruation	1= Once a day<br>2= Twice or more per day<br>3= Only the first day<br>4= I don't wash myself
46	Where does your household normally obtain drinking water?	1= Piped tap<br>2=Tube well or borehole<br>3=Protected well<br>4=Unprotected well<br>5=Protected spring<br>6=Unprotected spring<br>7=Rainwater<br>8=Tanker truck<br>9=Cart with small tank<br>10=Surface water (river, dam, lake, pond, or stream, canal, irrigation channel)

		11= Bottled water<br>12= Other
47	Where is the primary water source located?	1= In the house<br>2 = In the yard<br>3= At a relative's neighbor's house or yard<br>4= At a public location
48	51 a. Where did you defecate more often during Menstruation?	1=Facility in house or yard<br>2=Facility in relative or Neighbor's house or yard<br>3= Facility in community<br>4= Open space(bush/ field/Pond)
49	52 a. Where do you urinate more often during Menstruation?	1=Facility in house or yard<br>2=Facility in relative or Neighbor's house or yard<br>3= Facility in community<br>4= Open space(bush/ field/Pond)
50	53 a. How many minutes does it take for you to get to your primary defecation site during?	
51	54 a. How far is the water source you use when going to defecation during menstruation?	
52	Do you need to carry extra water when you go for urination when menstruating?	1=yes 0=no
53	Do you need to carry extra water when you go for defecation when menstruating?	1=yes 0=no
54	Is there a toilet facility in your household?	1=yes 0=no
55	How many years you had access to a latrine at your household?	
56	Do you use more the latrine during mensuration?	1=yes 0=no
57	Do you find enough privacy in the latrine you use?	1=yes 0=no
58	Does your latrine have a roof?	1=yes 0=no
59	Does your latrine have a door?	1=yes 0=no
60	Do you have a hand-washing facility inside/or close to your latrine?	1=yes 0=no
61	Do you have a disposal place (bucket, container or pit) inside /or close to your latrine?	1=yes 0=no
62	Where do you get your water for latrine use?	1= There is a tap inside the latrine<br>2= I bring it from my private tube well<br>3= I bring it from a neighbor tube well within 1 min of my home<br>4= I bring it from a public source within 1 min of my home<br>5= I bring it from a far source (more than 5min walk)
63	How do you normally wash your hands?	1= Water Only<br>2= Water and soap or detergent<br>3= Water and ashes<br>4= Water and soil/dirt/mud
64a	Do you usually wash your hands Before eating?	1=yes 0=no
64b	Do you usually wash your hands after eating?	1=yes 0=no
64c	Do you usually wash your hands Before cooking?	1=yes 0=no
64d	Do you usually wash your hands after handling domestic animals?	1=yes 0=no
64e	Do you usually wash your hands After defecating?	1=yes 0=no
64f	Do you usually wash your hands After urinating?	1=yes 0=no
64g	Do you usually wash your hands After cleaning child that defecate?	1=yes 0=no
64h	Do you usually wash your hands after changing menstrual hygiene pad?	1=yes 0=no
64i	Do you Never wash your hands?	1=yes 0=no

Study two Questionnaire

	Prompt	*Values*
1	Participant id	
2	Location	
3	Date	
4	Age: How old are you?	
5	Age category	1=18-25<br>2=26-35<br>3=36-45
6	What is your marital status	1=Single, never married<br>2=Married<br>3=Widowed/Divorced/Separated
7	How many years have you been married?	
8	Marry category	0=0 years<br>1=1-10<br>2=11-20<br>3=21 & above
9	Have you given birth within the last three months	1=yes 0=no
10	What is your religion?	1= Hindu<br>2= Muslim<br>3=Christian
11	What is your caste or tribe?	1= Scheduled caste (SC)<br>2= Scheduled tribe (ST)<br>3 =Other backward caste (OBC)<br>4= General<br>99= na
12	What is the highest standard year of education that you have completed?	1=No formal education<br>2=1-10th year<br>3=+2 year & above
13	What is your occupation?	1=Employed<br>2=self-employed<br>3= Housewife<br>4=Student<br>5=Unemployed
14	How many people do they live in your house?	
15	Family category	1=1-2<br>2=3-5<br>3=6 & above
16	Which is the monthly household income	1=Below 5,000<br>2=5,000-20,000<br>3=21,000-and above<br>4=Don't know
17	BPL card	1=yes 0=no
18	Where do you live	1=Private home in housing colony<br>2=Rented home in housing colony<br>3=Urban slum<br>4=Rural/village<br>5=Hostel
19	Do you have abnormal vaginal discharge	1=yes 0=no
20	Is the discharge excessive	1=yes 0=no

21	What is the color of the vaginal discharge	1=Yellow<br>2=White<br>3=Grey White<br>4=Green<br>99= na
22	What is the consistency of the vaginal discharge	1=Rare<br>2=Thick<br>99= na
23	What is the smell of the vaginal discharge	1=Unpleasant<br>2=Without smell<br>99= na
24	Do you have a feeling of burning or itching in the genitalia	1=yes 0=no
25	Do you present genital sores	1=yes 0=no
26	Do you have pain during sex	1=yes 0=no
27	Do you have a feeling of burning or itching or pain when urinating	1=yes 0=no
28	Do you have cloudy urine or blood in your urine?	1=yes 0=no
29	Symptomatic/Asymptomatic	1=yes 0=no
30	Do you have pain in your lower back?	1=yes 0=no
31	Do you have pain in your belly or stomach	1=yes 0=no
32	Do you have pain in your breast?	1=yes 0=no
33	Do you present abnormal vaginal bleeding out of the menstruation days	1=yes 0=no
34	When was the last time that you had your period?	
35	Do you use any type of Contraceptive method at the moment	1=yes 0=no
36	If you use a contraceptive method, choose which one	1=Condom use<br>2=Injections or birth control pills<br>3=Intrauterine device<br>4=Tubal ligation<br>9=na
37	Have you taken any antibiotic treatment during the last 2 weeks	1=yes 0=no
38	Is the problem why you are coming to the clinic a recurrent one	1=yes 0=no
39	How often have these problems appeared in the **last year** approximately?	1=Less than 3 times<br>2= More than 3 times<br>3=I don't remember<br>99=na
40	Did you change your menstrual hygienic habits after having this recurrent symptom?	1=yes 0=no
41	If yes, what did you change	1=Menstrual absorbent type<br>2=Vaginal washing practice<br>3=Body washing practices<br>4=Absorbent washing practices (if applied).<br>5=Place for changing menstrual absorbent.<br>6=Place to defecate or urinate every day<br>7= Place to defecate or urinate during<br>menstruation<br>99=na
42	Did you withhold defecation during menstruation?	1=yes 0=no
43	How old were you when you had your first period?	
44	How often do you get your period	1=Every 21 days or less<br>2=Every 21-26 days<br>3=Every 26-28 days<br>4=More than 30 days
45	For how long your period last?	1=3 days or less<br>2=4-5 days<br>3=6 days or more

46	How do you describe your period in terms of amount of blood loss during it?	1=Heavy<br>2=Moderate<br>3=Scanty
47	What was the most commonly absorbent material used during the last 6 cycles	1=Disposable sanitary pads<br>2=Reusable cloths/towel<br>3=Mixture of reusable and disposable<br>4=Nothing
48	What type of reusable material do you use	1=Old cotton fabric (sari or other)<br>2=Old silk/nylon fabric (sari or other)<br>3=Towel<br>99=na
49	Do you use underpants	1=Never use<br>2=Regularly use<br>3=Only during menstruation<br>4=Stop using during menstruation
50	How do you feel use of under pant during menstruation	1=Comfort<br>2=Discomfort
51	How often do you change the absorbent material in one of your heavier days?	1=Once a day<br>2=Twice a day<br>3=Three times a day or more
52	Where do you normally change your absorbent material?	1=Inside the toilet facility<br>2=Outside the toilet facility
53	Which season do you feel more challenges at your changing place	1=No season<br>2=Rainy season<br>3=Summer season<br>4=Winter season
54	If you reuse it, where do you wash it?	1=Inside the latrine or bathroom stall<br>2=At the tube well or yard<br>99=na
55	How do you wash your sanitary cloths?	1=With water and soap/detergent (Dettol)<br>2=With water only
56	How many times per day do you wash reusable absorbents	1=Once a day<br>2=Twice a day or more 99=na
57	Which season do you feel more challenges at washing your reusable absorbents?	1=No season<br>2=Rainy season<br>3=Summer season<br>4=Winter season<br>99=na
58	After washing it, how do you dry the cloth?	1=Dry it in the sun or open space<br>2=Dry it inside the house<br>3=I don't dry it<br>99=na
59	What problems do you face while drying reusable absorbents	1=Lack of privacy at drying place<br>2=Availability of time/family interference<br>3=Lack of space for drying<br>4=Insect or other animals urinate during drying<br>5=Neighborhood persons stole the clothes<br>99=na

60	How do you store the cloth for use next time	1=Wrapped in polythene/paper/container<br>2=Without wrapping<br>99=na
61	Where do you store the cloth for use next time?	1=In the cupboard/selves along with other clothes<br>2=In some place in the latrine or bathroom<br>99=na
62	Where do you dispose your sanitary pad	1=Inside latrine<br>2=Throw in the household garbage bin<br>3=Throw in garbage dump<br>4=Put it in the pond<br>5=Discard in any other open space
63	Which season do you feel more challenges at storing your reusable absorbents	1=No season<br>2=Rainy season<br>3=Summer season<br>4=Winter season<br>99=na
64	What type of washing (bath or vaginal wash) do you practice during menstruation	1=Only vaginal wash<br>2=Bath of full body
65	How often do you wash yourself (bath or vaginal wash) during menstruation?	1=Once a day<br>2=Twice or more per day
66	Where does your household normally obtain drinking water	1=Piped tap<br>2=Tube well or borehole or protected well<br>3=Unprotected well
67	Where is the primary water source located	1=In the house<br>2=In the yard<br>3=At a relative's or neighbor's house or yard<br>4=At a public location
68	Is there a latrine facility in your household	1=yes 0=no
69	Do you use your latrine	1=yes 0=no
70	Where do you normally go for defecation	1=Latrine facility inside house or yard<br>2=Latrine facility outside house or yard
71	Where do you defecate more often during menstruation	1=Latrine facility inside house or yard<br>2=Latrine facility outside house or yard
72	Where do you normally go for urination?	1=Latrine facility inside house or yard<br>2=Latrine facility outside house or yard
73	Where do you urinate more often during menstruation?	1=Latrine facility inside house or yard<br>2=Latrine facility outside house or yard
74	Do you use your latrine more often when you are menstruating	1=yes 0=no
75	Do you find enough privacy in the latrine/bathroom you use?	1=yes 0=no
76	Does your latrine/bathroom have a roof?	1=yes 0=no
77	Does your latrine/bathroom have a door?	1=yes 0=no
78	Can your latrine/bathroom be locked?	1=yes 0=no
79	Do you have a hand-washing facility inside/or close to your latrine?	1=yes 0=no
80	Do you have a disposal place (bucket, container or pit) inside /or close to your latrine?	1=yes 0=no

Supplementary File 2

Table S2. Associations with being below the poverty line (n=509)

Variables of interest	OR	95% CI	p-value (LRT)
**Education**			0.05
No formal education	1		

Primary or secondary	0.8	0.5-1.2	
Any level of higher education	0.5	0.3-0.9	
**Household latrine**			<0.01
No	1		
Yes, without privacy	0.6	0.3-1.0	
Yes, with privacy	0.3	0.2-0.5	
**Frequency of changing absorbents**			0.1
Once per day	1		
Twice per day	0.7	0.5-1.1	
Three or more times per day	0.6	0.4-1.0	
**Type of body washing during menstruation**			0.57
Vaginal wash only	1.0		
Full body bath	0.9	0.6-1.3	
**Frequency of washing**			0.56
Once per day	1.0		
Twice or more per day	0.9	0.7-1.3	
**Location of washing absorbent**			0.44
Latrine/bathroom area	1		
Tube well, public pond or river	1.1	0.8-1.6	
**Location of drying absorbents**			0.03
Sun or open space	1		
Inside the house	1.5	1.0-2.1	
**Location of absorbent storage between cycles**			0.26
Cupboard/shelves with other clothes	1		
Latrine or bathroom	1.2	0.9-1.7	
